# Monoaminergic and Kynurenergic Characterization of Frontotemporal Dementia and Amyotrophic Lateral Sclerosis in Cerebrospinal Fluid and Serum

**DOI:** 10.1007/s11064-020-03002-5

**Published:** 2020-03-04

**Authors:** Jana Janssens, Yannick Vermeiren, Martijn van Faassen, Claude van der Ley, Ido P. Kema, Peter P. De Deyn

**Affiliations:** 1 Department of Biomedical Sciences, Neurochemistry and Behaviour, Institute Born-Bunge (IBB), University of Antwerp, Wilrijk, Antwerp, Belgium; 2 Department of Neurology, Alzheimer Center Groningen, University Medical Center Groningen (UMCG) and University of Groningen, Hanzeplein 1, 9713 GZ Groningen, The Netherlands; 3 Department of Laboratory Medicine, University Medical Center Groningen (UMCG) and University of Groningen, Groningen, The Netherlands; 4 Faculty of Medicine and Health Sciences, University of Antwerp, Wilrijk, Antwerp, Belgium

**Keywords:** Biomarker, Neuropathology, Dementia, Neurophysiology

## Abstract

Exploring the neurochemical continuum between frontotemporal dementia (FTD) and amyotrophic lateral sclerosis (ALS) with respect to monoamines and kynurenines in cerebrospinal fluid (CSF) and serum, may be useful to identify possible new research/therapeutic targets. Hence, we analysed monoamines and kynurenines in CSF and serum derived from patients with FTD (n = 39), ALS (n = 23), FTD-ALS (n = 4) and age-matched control subjects (n = 26), using reversed-phase ultra-high performance liquid chromatography (RP-UHPLC) with electrochemical detection (ECD) and liquid chromatography tandem mass spectrometry, respectively. We noted a shared dopaminergic disturbance in FTD and ALS when compared to CONTR, with significantly increased serum DA levels and decreased DOPAC concentrations, as well as decreased DOPAC/DA ratios in both disease groups. In CSF, significantly reduced DOPAC concentrations in FTD and ALS were observed as well. Here, a significant increase in DA levels and decrease in DOPAC/DA ratios was only found in FTD relative to CONTR. With respect to the kynurenine pathway (KP), we only found decreased HK/XA ratios, indicative for vitamin B6 status, in serum of ALS subjects compared to FTD. The dopaminergic commonalities observed in FTD and ALS might relate to a disturbance of dopaminergic nerve terminals in projection areas of the substantia nigra and/or ventral tegmental area, although these findings should first be confirmed in brain tissue. Lastly, based on the results of this work, the KP does not hold promise as a research/therapeutic target in FTD and ALS.

**Electronic supplementary material:**

The online version of this article (10.1007/s11064-020-03002-5) contains supplementary material, which is available to authorized users.

## Introduction

Frontotemporal dementia (FTD) and amyotrophic lateral sclerosis (ALS) are devastating neurodegenerative disorders demonstrating genetic and neuropathologic overlap. The most common shared genetic mutation is a GGGGCC expansion in *C9ORF72*, present in approximately 30–47% [1, 2] and 25–34% [1, 3] of familial ALS and FTD cases, respectively. This hexanucleotide expansion is most often associated with the presence of cytoplasmic inclusions containing transactive response DNA-binding protein of 43 kDa (TDP-43) in both diseases [4, 5]. Consequently, these inclusions are found in up to 97% of ALS and 45% of FTD cases [6]. Next to the shared genetic and neuropathologic characteristics, similarities in clinical presentation are also observed. Behavioural abnormalities and/or signs of cognitive impairment including executive dysfunction, are observed in 50% of patients with ALS, while 15% of these subjects reach the diagnostic criteria for FTD [7–10]. Likewise, similar frequencies of motor disturbances are observed in FTD [11, 12].

Although this clinicopathologic overlap has been well covered in the scientific literature, less is known about (monoaminergic) neurotransmitter disturbances in ALS. As this condition is currently treated with strategies primarily targeting glutamatergic neurotransmission and oxidative stress using riluzole and edaravone, respectively [13–15], offering limited benefit, other options based on alternative neurotransmitter systems could be explored. The relative success of selective serotonin reuptake inhibitors (SSRIs) in FTD might point to monoamines as interesting research targets in this disorder, and possibly also in ALS. Furthermore, as previous research demonstrated monoaminergic disturbance in brain tissue (reviewed in [16]) and cerebrospinal fluid (CSF) of FTD patients [17–19], investigating the occurrence of these substances in easily accessible body fluids such as blood, plasma or serum in ALS might be of interest to determine whether FTD and ALS also share neurochemical characteristics.

Due to its implication in (neuro-)inflammation, the kynurenine pathway (KP) has received increasing attention in immune-related diseases such as cancer [20, 21], HIV [22], and several neurodegenerative disorders including ALS [23–25]. Although few researchers have previously focused on determining the KP in samples derived from patients with ALS, it appears that concentrations of some neuroprotective and neurotoxic metabolites are altered in CSF and serum [23, 26]. In FTD, however, little information regarding a possible dysregulation of this metabolic pathway can be found. Given the involvement of the KP in neuroinflammation and since this pathway is linked to the production of 5-HT via their common precursor l-tryptophan (TRP), not only 5-HT and other related monoamines, but also compounds of the KP could possibly represent an interesting means to investigate the neurochemical continuum of FTD-ALS.

## Materials and Methods

### Study Population

**Fig. 1:**
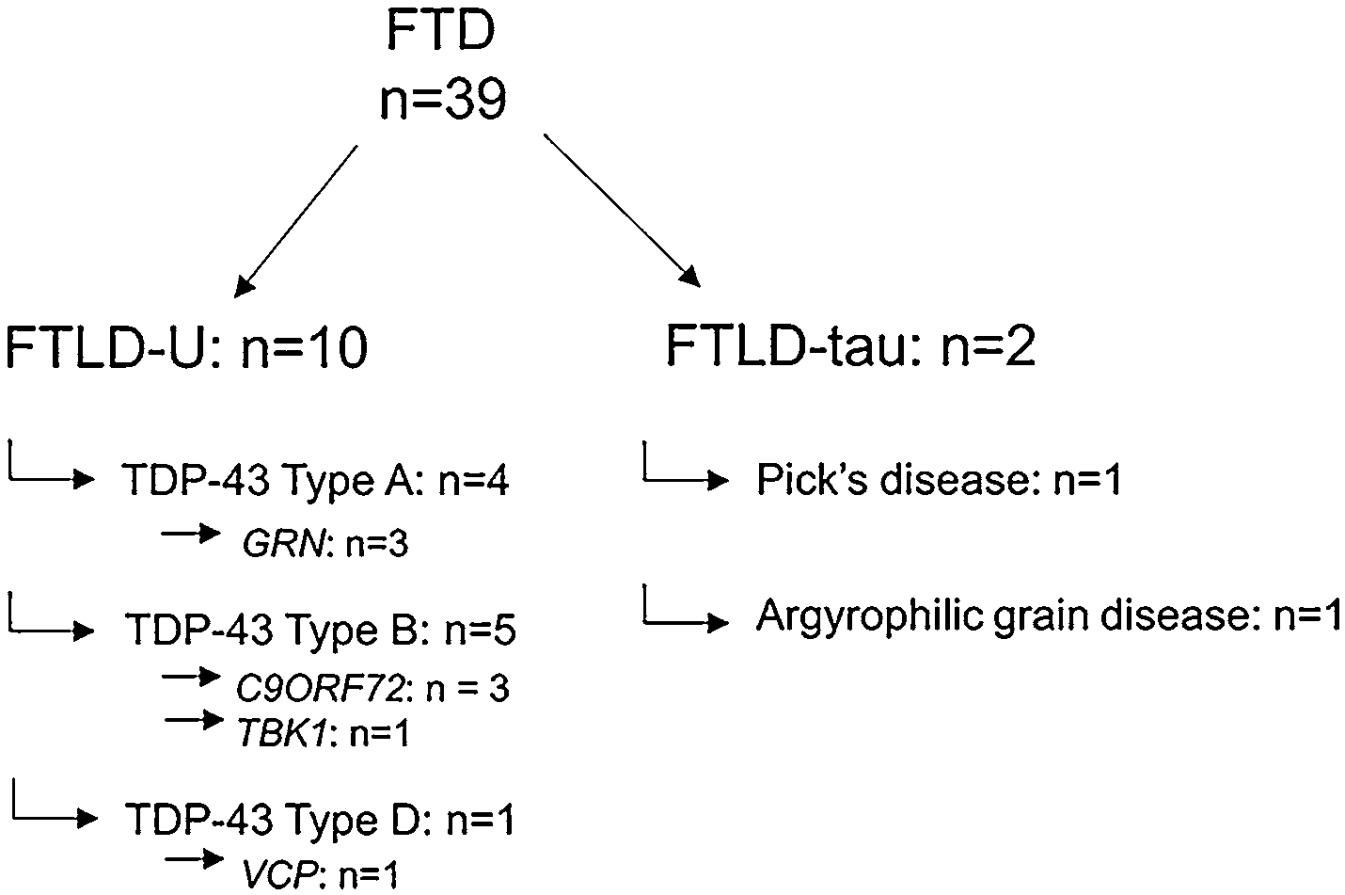
Overview of neuropathological diagnoses in the FTD group. *C9ORF72* chromosome 9 open reading frame 72, *FTLD-U* frontotemporal lobar degeneration with ubiquitin-positive inclusions, *FTD* frontotemporal dementia, *GRN* progranulin gene, *TBK1* TANK-binding kinase 1, *TDP-43* transactive response DNA-binding protein of 43 kDa, *VCP* valosin-containing protein

This retrospective study used CSF and serum samples derived from the NeuroBioBank of the Institute Born-Bunge (NBB-IBB (no. BB190113), Wilrijk). Monoamines and metabolites were analysed in CSF samples of patients suffering from probable FTD (n = 39) [27], FTD-ALS (n = 4), suspected, possible and probable ALS (n = 23) [28], and age-matched control subjects (CONTR) (n = 26). Serum samples were derived from the same patients, although for 3 ALS subjects, no serum samples were available. Patients and control subjects were recruited at the Memory Clinic of the Hospital Network Antwerp (ZNA) Middelheim and Hoge Beuken, as CSF and serum sampling was part of their clinical diagnostic workup. Psychiatric antecedents and central nervous system pathology other than FTD and ALS were regarded as exclusion criteria. Control subjects came to the clinic because of (tension) headache, lumbar canal stenosis, cervicalgia/cervical myelopathy, carpal tunnel syndrome, nausea, chronic gait disorders, periodic fever syndrome, leucopenia, struma simplex, facial arteriovenous malformations, and sinusitis. In patients who gave brain donation consent, brain autopsy was generally performed within 6–8 h postmortem. The right hemisphere was immersion-fixated in a 12% formalin solution for neuropathological examination, while the left hemisphere was frozen at – 80 °C [29, 30]. In the FTD-ALS group, only one subject had neuropathological confirmation of the initial clinical diagnosis, with molecular lesions classified as TDP-43 Type B. Similarly, only one subject was neuropathologically confirmed as definite ALS. Genetically, 11 FTD subjects had a hexanucleotide expansion in *C9ORF72*, three patients had a *GRN* mutation, two subjects had a *MAPT* mutation and two other patients had a mutation in either *TBK1* or *VCP*. A summary of neuropathologically confirmed cases and related genetics in the FTD group can be found in Fig. 1. This study was approved by the Medical Ethical Committee of the Middelheim General Hospital (Antwerp, Belgium, file numbers 2805 and 2806), and was conducted in line with the Helsinki Declaration.

### Sampling Of Cerebrospinal Fluid And Serum

Lumbar puncture was performed at the L3/L4 or L4/L5 intervertebral space to collect a total volume of 16.5 mL, which was divided across five fractions of 4.5 mL, 1.5 mL, 1.5 mL, 4.5 mL and 4.5 mL, respectively, in polypropylene vials (Nalgene; VWR, Leuven, Belgium) [31]. Serum was obtained after total blood sampling into two serum gel tubes with clotting activator (S-Monovette 7.5 mL Z-gel (Sarstedt, Nümbrecht, Germany)), which were centrifuged during 10 min at 3000 rpm. Afterwards, serum was distributed to polypropylene vials. Both CSF and serum samples were frozen and stored at – 80 °C until analysis.

### RP-UHPLC-ECD

An optimized and validated reversed-phase ultra-high-performance liquid chromatography (RP-UHPLC) system with electrochemical detection (ECD) was used to determine (nor)adrenaline and its metabolite 3-methoxy-4-hydroxyphenylglycol (MHPG), dopamine (DA) and its metabolites 3,4-dihydroxyphenylacetic acid (DOPAC) and homovanillic acid (HVA), as well as 5-HT and its metabolite 5-hydroxyindoleacetic acid (5-HIAA). The sample preparation consisted of a purification on Amicon® Ultra 0.5 Centrifugal Filters (cutoff 3000 Da; Millipore, Ireland), which were washed twice beforehand with 450 µL buffer while centrifuging (14,000×*g*, 25 min, 4 °C). Serum and CSF were brought onto these columns, followed by centrifugation at 4 °C for 40 min at 14,000×*g*. The resulting CSF filtrate was diluted 1:2, and either 1:7 or 1:10, while the filtered serum was diluted 1:5 and 1:25 or 1:30. This RP-UHPLC system operated at an isocratic flow rate of 75 μL/min. The Decade II electrochemical detector was equipped with a thin layered electrochemical VT03 flow cell fitted with a glassy carbon 0.7 mm working electrode and an in situ Ag/AgCl (ISAAC) reference electrode. Samples of 5 μL were loaded with an Alexys AS 110 Autosampler. Separation was achieved using a short 15 cm Acquity Column (BEH C18, 1 mm diameter, particle size 1.7 μm; Waters, Etten-Leur, the Netherlands) [32].

### LC–MS/MS

Concentrations of TRP, l-kynurenine (KYN), 3-hydroxykynurenine (HK), anthranilic acid (AA), kynurenic acid, xanthurenic acid (XA), quinolinic acid, picolinic acid, and nicotinic acid, were determined at the department of Laboratory Medicine of the University Medical Center Groningen by liquid chromatography in combination with isotope dilution tandem mass spectrometry (LC–MS/MS) essentially as described by [33]. In short, 100 µL of CSF or 50 µL of serum was mixed with deuterated or ^13^C-labeled internal standards of the KP compounds under investigation. These mixtures were extracted with Strata X-A 96-well plates with a pore size of 33 µm and sorbent mass of 30 mg/well (Cat. No.: 8E-S123-TGB; Strata-X, Phenomenex, Utrecht, the Netherlands) and eluted with 3 M HCl in 1-butanol. Finally, 1 µL was injected into an Acquity UHPLC (Waters, Etten-Leur, the Netherlands) equipped with a Phenomenex Luna column (Omega C18, 100 × 2.1 mm, particle size 1.6 µm), and coupled to a XEVO TQ-S MS/MS system (Waters, Etten-Leur, the Netherlands). KYN/TRP ratios were calculated as a measure for TDO and IDO activity [34] and HK/XA ratios as a measure for vitamin B6 function since the enzyme converting HK to XA, kynurenine aminotransferase II, is pyridoxal phosphate-dependent [35].

### Statistics

One-way ANOVA was applied for continuous demographic parameters such as age and storage time. In case the assumption of homogeneity of variances was violated, Welch’s ANOVA was applied. Fisher’s Exact tests were applied to investigate the association between categorical variables such as disease category, gender and medication use.

Nonparametric statistics were applied to test inter-group differences of monoamines and kynurenines due to the non-normal distribution of these parameters in CSF and serum. Kruskal–Wallis analyses were applied as omnibus tests, while post-hoc analyses were performed with Mann–Whitney *U* statistics. Correction for multiple testing was applied using the Benjamini–Hochberg procedure. The results for the FTD-ALS group are presented solely as median concentrations, as the size of this group was very small and often even reduced to n = 2 or n = 3. Therefore, we opted not to include monoaminergic and kynurenergic concentrations of this group in statistical tests for group comparisons. Lastly, Spearman’s rank correlation analysis was used to assess the relationship between storage time and concentrations of monoamines and kynurenines. Again, Benjamini–Hochberg corrections were applied to account for multiple testing. All statistical analyses were performed using SPSS version 25.0 for Windows. Figures were created with GraphPad Prism version 6 for Windows (GraphPad Software, La Jolla California USA, www.graphpad.com).

## Results

### Demographics

Table 1 summarizes the demographic details of the study population. Additional information about the types of medication across the different disease groups can be found in Online Resource 1.

**Table 1:** Demographics of the study populations for analysis of monoamines and kynurenines

Parameter	Compounds of interest	Sample type	CONTR	FTD	FTD-ALS	ALS	Test statistic
Age at sampling (years)	Monoamines	CSF	67.0 ± 8.0 n = 26	67.4 ± 11.6 n = 39	64.7 ± 5.7 n = 3	67.2 ± 10.3 n = 23	F(3,87) = 0.069 *P* > 0.05
		Serum	67.2 ± 8.3 n = 26	67.4 ± 11.6 n = 39	64.7 ± 5.7 n = 3	66.9 ± 10.4 n = 20	F(3,84) = 0.071 *P* > 0.05
	Kynurenines	CSF	67.3 ± 8.1 n = 25	67.4 ± 11.6 n = 39	64.7 ± 5.7 n = 3	67.6 ± 10.5 n = 22	F(3,85) = 0.071 *P* > 0.05
		Serum	67.0 ± 8.0 n = 26	67.4 ± 11.6 n = 39	64.7 ± 5.7 n = 3	66.9 ± 10.4 n = 20	F(3,84) = 0.072 *P* > 0.05
Male/female	Monoamines	CSF	14/12	20/19	1/3	21/2	FE = 14.2; *P* < 0.05
		Serum	14/12	20/19	1/2	18/2	FE = 10.9; *P* < 0.05
	Kynurenines	CSF	14/11	20/19	1/3	20/2	FE = 13.1; *P* < 0.05
		Serum	14/12	20/19	1/2	18/2	FE = 10.9; *P* < 0.05
Taking/not taking medication	Monoamines	CSF	9/7	27/7	0/1	11/6	FE = 5.2 *P* > 0.05
		Serum	10/6	27/7	0/1	11/5	FE = 4.1 *P* > 0.05
	Kynurenines	CSF	9/7	27/7	0/1	11/6	FE = 5.2 *P* > 0.05
		Serum	9/7	27/7	0/1	11/5	FE = 5.1 *P* > 0.05
Storage time (months)	Monoamines	CSF	202.5 ± 30.6 n = 26	132.8 ± 62.1 n = 39	158.3 ± 44.4 n = 3	171.9 ± 76.2 n = 23	Welch = 10.930 *P* < 0.05
		Serum	203.0 ± 30.6 n = 26	133.2 ± 62.2 n = 39	158.3 ± 44.4 n = 3	187.3 ± 68.2 n = 20	Welch = 10.848 *P* < 0.05
	Kynurenines	CSF	213.0 ± 30.8 n = 25	144.6 ± 62.2 n = 39	169.7 ± 44.2 n = 3	185.9 ± 77.3 n = 22	Welch = 10.285 *P* < 0.05
		Serum	214.2 ± 30.7 n = 26	144.6 ± 62.1 n = 39	169.7 ± 44.2 n = 3	198.8 ± 68.0 n = 20	Welch = 10.760 *P* < 0.05

Data regarding age at sampling and sampling time are represented as mean ± standard deviation. For the numbers of subjects taking/not taking medication, psychotropic substances, riluzole and dietary vitamin B supplements were taken into account

*ALS* amyotrophic lateral sclerosis, *CONTR* control, *CSF* cerebrospinal fluid, *FE* Fisher’s Exact, *FTD* frontotemporal dementia, *FTD-ALS* frontotemporal dementia—amyotrophic lateral sclerosis

### Monoamines

**Fig. 2:**
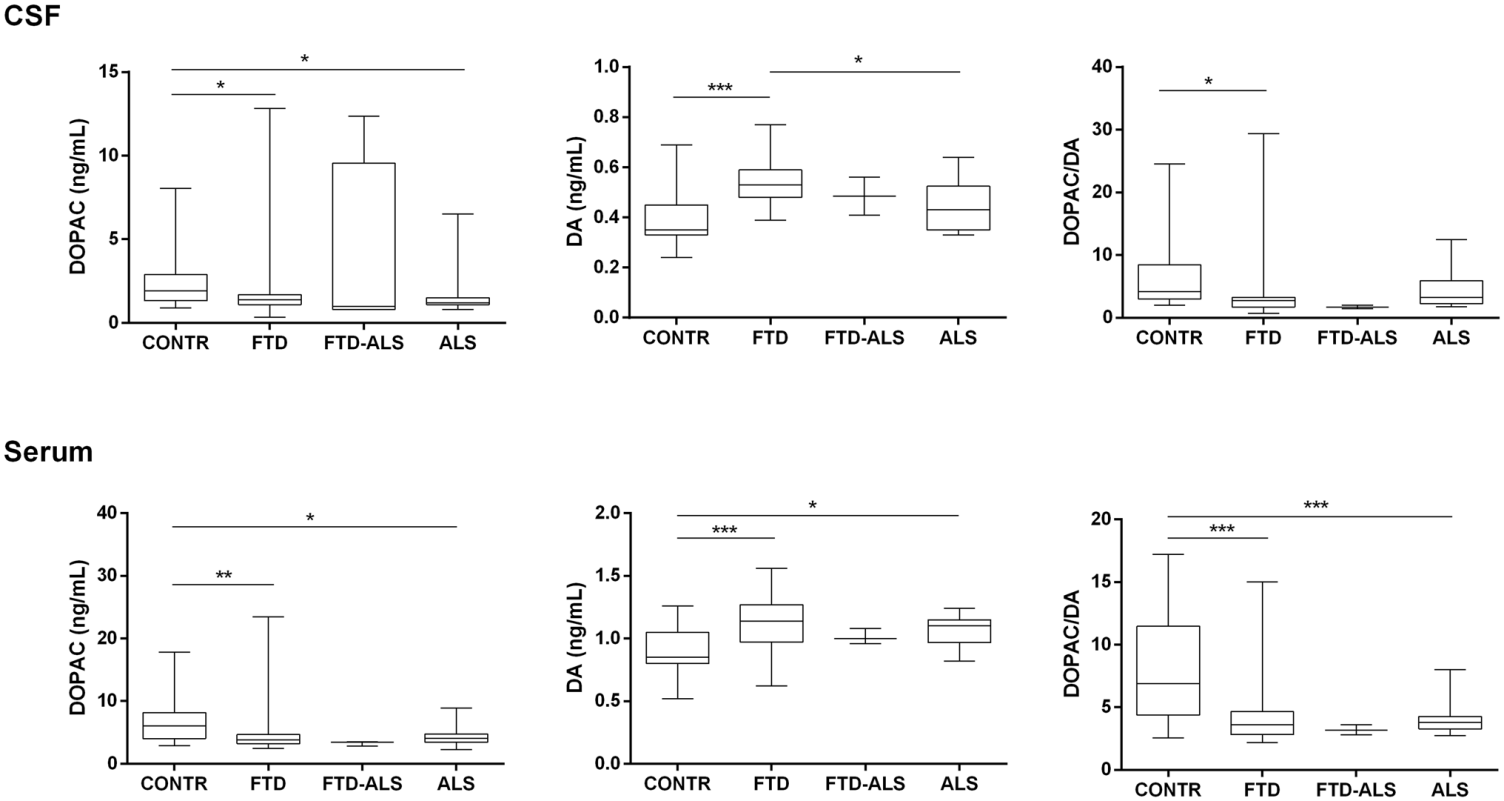
Dopaminergic findings across diagnostic categories. Data are represented as box- and whisker plots with minimum–maximum ranges. Statistically significant differences after Mann–Whitney *U* tests with Benjamini–Hochberg post-hoc corrections are depicted by one, two or three asterisks if *P* ≤ 0.05, 0.001, or 0.0001, respectively. Sample sizes for CSF DOPAC are: CONTR: n = 26, FTD: n = 39, FTD-ALS: n = 4, ALS: n = 23, sample sizes for CSF DA and DOPAC/DA are CONTR: n = 21, FTD: n = 35, FTD-ALS: n = 2, ALS: n = 17. Sample sizes for serum DOPAC are: CONTR: n = 26, FTD: n = 39, FTD-ALS: n = 3, ALS: n = 20, sample sizes for serum DA and DOPAC/DA are: CONTR: n = 26, FTD: n = 35, FTD-ALS: n = 3, ALS: n = 20. The FTD-ALS group was not included in the statistical analysis. *ALS* amyotrophic lateral sclerosis, *CONTR* control, *CSF* cerebrospinal fluid, *DA* dopamine, *DOPAC* 3,4-dihydroxyphenylacetic acid, *FTD* frontotemporal dementia, *FTD-ALS* frontotemporal dementia—amyotrophic lateral sclerosis

**Fig. 3:**
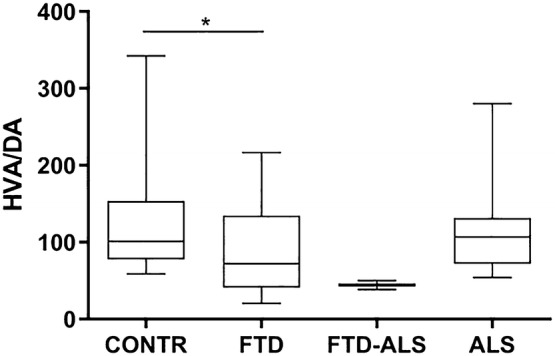
CSF HVA/DA ratios across diagnostic categories. Data are represented as box- and whisker plots with minimum–maximum ranges. Statistically significant differences after Mann–Whitney *U* analyses with Benjamini–Hochberg corrections are indicated by an asterisk (*P* < 0.05). Sample sizes for CSF HVA/DA are: CONTR: n = 21, FTD: n = 35, FTD-ALS: n = 2, ALS: n = 17. The FTD-ALS group was not included in the statistical analysis. *ALS* amyotrophic lateral sclerosis, *CONTR* control, *CSF* cerebrospinal fluid, *DA* dopamine, *FTD* frontotemporal dementia, *FTD-ALS* frontotemporal dementia-amyotrophic lateral sclerosis, *HVA* homovanillic acid

Alterations in the dopaminergic system across disease groups were noted in both CSF (H(2) = 9.017, *P* < 0.05 for DOPAC; H(2) = 17.963, *P* < 0.001 for DA; H(2) = 12.392, *P* < 0.05 for DOPAC/DA and H(2) = 7.252, *P* < 0.05 for HVA/DA) and serum (H(2) = 12.116, *P* < 0.05 for DOPAC, H(2) = 16.850, *P* < 0.001 for DA; and H(2) = 19.438, *P* < 0.0001 for DOPAC/DA). A general decrease in DOPAC values was observed in FTD and ALS subjects compared to the CONTR group. Increased DA concentrations in CSF were found in the FTD versus the CONTR and ALS groups. In serum, DA levels were also highly significantly increased in the FTD subjects compared to CONTR individuals, while a similar result was observed between the CONTR and ALS group. Analysis of the DOPAC/DA ratio revealed a disturbed dopaminergic catabolism in FTD versus the CONTR group, evidenced by significantly lower CSF and serum DOPAC/DA ratios in the former category. Furthermore, serum DOPAC/DA ratios were severely decreased in ALS subjects compared to healthy CONTR subjects (Fig. 2). Another index of DA catabolism, CSF HVA/DA, was also significantly decreased in FTD compared to CONTR subjects (Fig. 3).

**Fig. 4:**
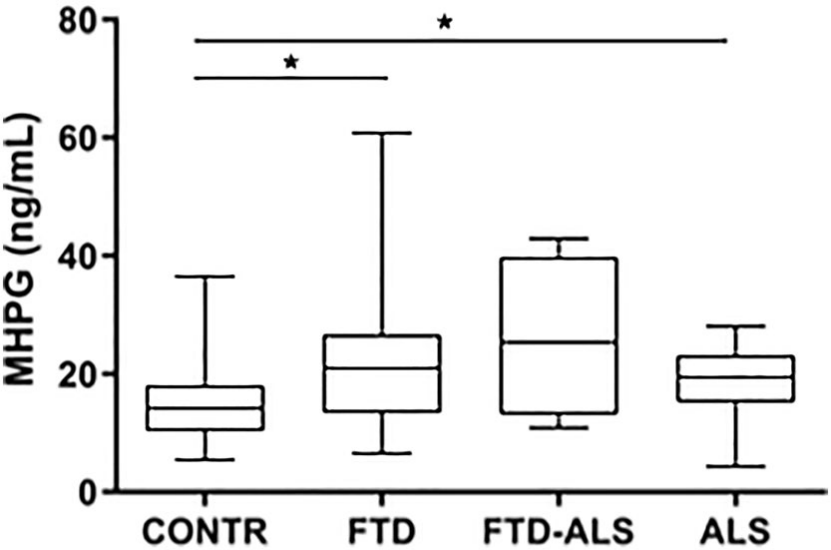
CSF MHPG levels across diagnostic categories. Data are represented as box- and whisker plots with minimum–maximum ranges. Statistically significant differences after Mann–Whitney *U* analyses with Benjamini–Hochberg corrections are indicated by an asterisk (*P* < 0.05). Sample sizes for CSF MHPG are: CONTR: n = 26, FTD = 39, FTD-ALS: n = 4, ALS: n = 23. The FTD-ALS group was not included in the statistical analysis. *ALS* amyotrophic lateral sclerosis, *CONTR* control, *CSF* cerebrospinal fluid, *FTD* frontotemporal dementia, *FTD-ALS* frontotemporal dementia-amyotrophic lateral sclerosis, *MHPG* 3-methoxy-4-hydroxyphenylglycol

Finally, CSF MHPG differed across diagnostic categories (H(2) = 8.833, *P* < 0.05), with significant differences after post-hoc correction indicating higher MHPG levels in FTD and ALS compared to the CONTR group (Fig. 4). All results of post-hoc comparisons are depicted in Online Resource 2.

### Kynurenines

**Fig. 5:**
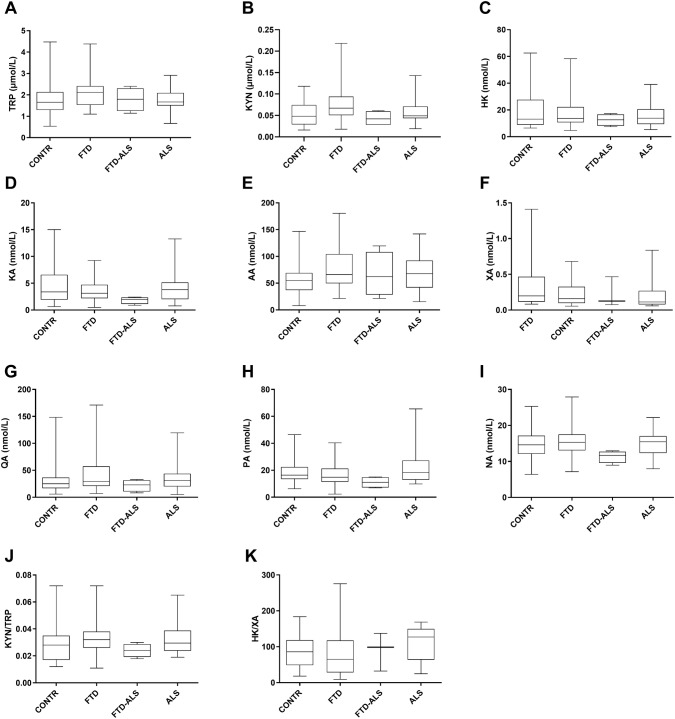
The KP in CSF. Data are represented as box- and whisker plots with minimum–maximum ranges. Statistically significant differences after Mann–Whitney *U* analyses with Benjamini–Hochberg corrections are indicated by an asterisk (*P* < 0.05). Sample sizes for TRP are: CONTR: n = 23, FTD: n = 37, FTD-ALS: n = 4, ALS: n = 20, sample sizes for KYN are: CONTR: n = 21, FTD: n = 37, FTD-ALS: n = 4, ALS: n = 21, sample sizes for HK are: CONTR: n = 24, FTD: n = 36, FTD-ALS: n = 4, ALS: n = 21, sample sizes for KA are: CONTR: n = 25, FTD: n = 39, FTD-ALS: n = 4, ALS: n = 22, sample sizes for AA are: CONTR: n = 24, FTD: n = 39, FTD-ALS: n = 4, ALS: n = 22, sample sizes for XA are: CONTR: n = 17, FTD: n = 30, FTD-ALS: n = 3, ALS: n = 18, sample sizes for QA are: CONTR: n = 23, FTD: n = 37, FTD-ALS: n = 4, ALS: n = 21, sample sizes for PA are: CONTR: n = 24, FTD: n = 39, FTD-ALS: n = 4, ALS: n = 22, sample sizes for NA are: CONTR: n = 25, FTD: n = 39, FTD-ALS: n = 4, ALS: n = 22, sample sizes for KYN/TRP are: CONTR: n = 21, FTD: n = 35, FTD-ALS: n = 4, ALS: n = 20, sample sizes for HK/XA are: CONTR: n = 15, FTD: n = 30, FTD-ALS: n = 3, ALS: n = 18. *AA* anthranilic acid; *ALS* amyotrophic lateral sclerosis, *CONTR* control, *CSF* cerebrospinal fluid, *FTD* frontotemporal dementia, *FTD-ALS* frontotemporal dementia-amyotrophic lateral sclerosis, *HK* 3-hydroxykynurenine, *KA* kynurenic acid, *KP* kynurenine pathway, *KYN*l-kynurenine, *NA* nicotinic acid, *PA* picolinic acid, *QA* quinolinic acid, *TRP*l-tryptophan, *XA* xanthurenic acid

**Fig. 6:**
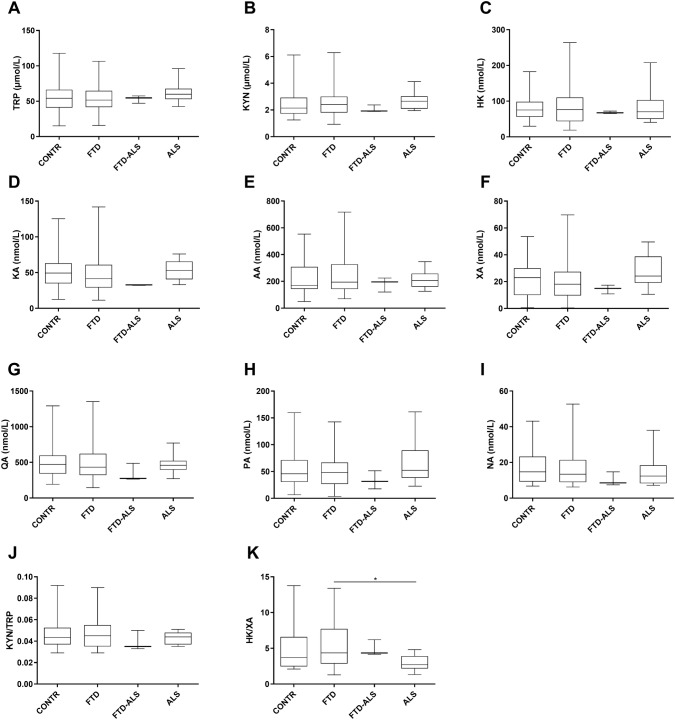
The KP in serum. Data are represented as box- and whisker plots with minimum–maximum ranges. Statistically significant differences after Mann–Whitney *U* analyses with Benjamini–Hochberg corrections are indicated by an asterisk (*P* < 0.05). Sample sizes for TRP are: CONTR: n = 26, FTD: n = 39, FTD-ALS: n = 3, ALS: n = 20, sample sizes for KYN are: CONTR: n = 26, FTD: n = 39, FTD-ALS: n = 3, ALS: n = 20, sample sizes for HK are: CONTR: n = 24, FTD: n = 39, FTD-ALS: n = 3, ALS: n = 19, sample sizes for KA are: CONTR: n = 26, FTD: n = 39, FTD-ALS: n = 3, ALS: n = 20, sample sizes for AA are: CONTR: n = 26, FTD: n = 38, FTD-ALS: n = 3, ALS: n = 20, sample sizes for XA are: CONTR: n = 25, FTD: n = 39, FTD-ALS: n = 3, ALS: n = 20, sample sizes for QA are: CONTR: n = 25, FTD: n = 37, FTD-ALS: n = 3, ALS: n = 18, sample sizes for PA are: CONTR: n = 25, FTD: n = 39, FTD-ALS: n = 3, ALS: n = 20, sample sizes for NA are: CONTR: n = 24, FTD: n = 38, FTD-ALS: n = 3, ALS: n = 20, sample sizes for KYN/TRP are: CONTR: n = 26, FTD: n = 39, FTD-ALS: n = 3, ALS: n = 19, sample sizes for HK/XA are: CONTR: n = 22, FTD: n = 38, FTD-ALS: n = 3, ALS: n = 18

No statistically significant differences were found after Kruskal–Wallis analysis for individual compounds belonging to the KP, except for CSF TRP (H(2) = 6.088, *P* < 0.05), however, intergroup comparisons for CSF TRP with post-hoc Mann–Whitney *U* tests did not remain significant following Benjamini–Hochberg corrections for multiple comparisons (Fig. 5). We also found a difference in serum HK/XA ratios across diagnostic categories (H(2) = 8.548, *P* < 0.05), with lower serum HK/XA ratios in the ALS versus the FTD group (Fig. 6). Post-hoc statistics, in addition to median values for HK/XA in each group, are summarized in Online Resource 2.

### Influence of Psychotropic Medication and Storage Time

When equivalent analyses were performed to detect differences in monoamine/kynurenine content only in subjects who were free of psychotropic medication and who did not take riluzole nor supplements containing vitamin B, a trend for increased CSF DA levels in FTD and ALS compared to CONTR could be observed (H(2) = 5.751, *P* = 0.056), but none of the monoaminergic differences remained statistically significant. In contrast, a significant difference was found for CSF TRP across groups (H(2) = 6.548, *P* < 0.05).

We found that CSF MHPG and DA were negatively correlated with storage time (rs(89) =  − 0.409, *P* < 0.0001 and rs(73) =  − 0.330, *P* < 0.05), whereas a positive association between storage time and 5-HIAA (rs(89) = 0.286, *P* < 0.05) as well as 5-HIAA/5-HT ratios (rs(87) = 0.346, *P* ≤ 0.001) was noted. In serum, negative correlations were noted between DA levels and storage time (rs(82) =  − 0.242, *P* < 0.05)). As for kynurenines, CSF TRP and serum HK/XA ratios decreased with increasing storage time (rs(81) =  − 0.232, *P* < 0.05 and rs(81) =  − 0.241, *P* < 0.05, respectively).

## Discussion

### Monoamines

A general dopaminergic disturbance was noted in FTD and ALS compared to CONTR subjects, largely corresponding to previous reports. Single photon emission computed tomography has shown decreased frontal uptake of a dopamine 2 receptor (D2) postsynaptic ligand, as well as a relative frontal hypoperfusion in FTD versus CONTR and subjects with Alzheimer’s disease [36]. Reduced uptake of ^11^C-2β-carbomethoxy-3β-(4-fluorophenyl) tropane (^11^C-CFT), a cocaine analogue targeting DA transporters (DAT), in the putamen of FTD subjects was also observed in another study [37]. In addition, ALS subjects showed decreased striatal binding of ^123^I-N-(3-iodopropen-2-yl)-2β-carbomethoxy-3β-(4-chlorophenyl)-tropane (^123^IPT), a cocaine analogue binding specifically to DATs [38]. Furthermore, increased DA levels and decreased HVA/DA ratios were observed in Brodmann area (BA)46 and BA6 of FTD subjects compared to CONTR [29]. Taken together, these findings may all indicate disrupted dopaminergic nerve terminals in projection areas of the substantia nigra (SN) and ventral tegmental area (VTA). Reduced presynaptic uptake of ^123^IPT and decreased binding of ^11^C-CFT in ALS and FTD, might reflect a dysfunction of dopaminergic nerve terminals, possibly related to presynaptic reuptake mechanisms mediated by DAT. This might explain our observation of increased CSF DA levels in these disease groups. The decreased DOPAC concentrations and DOPAC/DA ratios may then be a result of impaired DOPAC synthesis due to dopaminergic neuron dysfunction. Exploring the observed dopaminergic alterations in brain tissue of FTD and ALS subjects is mandatory to assess the validity of this hypothesis.

It would also be interesting to compare the dopaminergic disturbance in the FTD-ALS continuum with that observed in Parkinson’s disease (PD). In a previous study on dopaminergic alterations in CSF of synucleinopathy patients [39], among which PD, dopaminergic data of the PD group were reduced to eliminate influences of dopaminergic medication, resulting in a PD group (n = 17) in which only PD patients with CSF and plasma l-DOPA values ≤ 5 nmol/L and 10 nmol/L, were included. We calculated DA ratios and DOPAC ratios in CSF for the disease groups included in our study versus the control group (CONTR), as well as identical ratios for the PD group versus CONTR in the previous report [39]. We observed that the ratios of DOPAC in the FTD, FTD-ALS and ALS groups versus CONTR were 0.7, 0.5 and 0.6, respectively, while this ratio was 0.4 if the DOPAC concentrations of the PD group were normalized to CONTR. For DA, we noted ratios of 1.3 in both FTD as well as FTD-ALS groups, and 1.0 in ALS compared to CONTR. In the study on catecholaminergic alterations in synucleinopathy subjects [39], the PD/CONTR ratio for DA was 0.8.

In this respect, it is clear that the extent of the dopaminergic disturbance is most evident in PD, with severely decreased CSF DOPAC and DA concentrations compared to CONTR. This finding of lowered CSF DOPAC and DA concentrations in PD are supported by several other studies [40, 41], even though the sample sizes of these studies were rather small (n = 4 and n = 7, respectively). It is interesting to note that we did not find decreased DA concentrations in FTD or ALS, possibly reflecting a distinct disease mechanism from PD.

The dopaminergic alterations in serum of FTD and ALS versus CONTR subjects resembled those in CSF, which is surprising since DA nor DOPAC readily cross the blood–brain barrier (BBB) [42, 43]. However, several studies have demonstrated impaired BBB integrity in FTD and ALS [44, 45], which might account for the similarities observed in CSF and serum.

The use of psychotropic medication may have influenced our findings since the dopaminergic alterations could not be detected between FTD, ALS or CONTR in a medication-free subgroup. However, given the low number of observations in this analysis (n = 3–9 per group), type II errors cannot be ruled out.

The longer storage time in the CONTR group might have led to a false positive result regarding the increased MHPG and DA levels in FTD compared to the CONTR group. Thus, the absolute DA levels in CSF should be considered carefully, while the DOPAC concentrations and the dopaminergic turnover in CSF remain unaffected by storage time.

Based on the therapeutic strategies applied in FTD, we expected to find serotonergic (dis)similarities, in the ALS-FTD continuum. However, our results suggest a shared dopaminergic disturbance in CSF and serum, except for CSF DA levels. This finding may shift the research in these neurodegenerative conditions to DA-based approaches.

### Kynurenines

In previous studies investigating the KP in ALS, KYN levels, neuroprotective and neurotoxic KP compounds appeared to be altered in this condition [23, 26]. It is possible we could not reproduce these findings because of differences in CSF handling, alternative analytical conditions, or distinct demographics. Since the study of Chen et al. included non-age-matched CONTR and ALS groups (average age of 36 and 58 years, respectively) and since the KP is involved in aging, these results, particularly with respect to CSF KYN [46], KYN/TRP ratios as a measure for IDO-activity, and concentrations of QA [47] could also be explained by the higher average age in the ALS group.

The results of subjects who did not take psychotropic medication, riluzole or vitamin B supplements did indicate altered CSF TRP levels between FTD, ALS and CONTR. It is difficult to speculate about the (patho)physiological meaning of this result, as lowered KYN/TRP ratios would indicate a downregulation of IDO/TDO activity, but no such results could be observed. In addition, no serotonergic differences were observed in FTD and ALS versus the CONTR group, indicating that TRP metabolism was not shifted towards the synthesis of serotonergic compounds in these groups. Lastly, our observation of decreased serum HK/XA ratios in ALS compared to FTD could have been influenced by storage time. Another compound of the KP for which we found significant differences, CSF TRP, was also influenced by storage time. Based on the results of this study, we suggest that the KP offers limited therapeutic value/research potential for the FTD-ALS continuum.

### Future Perspectives

Although the SN was shown to be affected in FTD and ALS [48, 49], the differential pathology in the SN and/or VTA of FTD and ALS patients does not represent a well-studied topic. It would be interesting to investigate the correlation between neuropathological observations in the SN and VTA, and dopaminergic disturbance in their projection areas in postmortem brain tissue. Alternatively, resting state functional MRI may be applied to assess the functional connectivity between midbrain dopaminergic centers and (pre)frontal cortical regions, combined with CSF sampling in FTD and ALS subjects. Follow-up of these patients may then result in a better understanding of dopaminergic disturbance, and how this is reflected in bodily fluids. In addition, we recommend the analysis of CSF and serum on a clinically better characterized population, certainly with respect to the ALS group.

In conclusion, we found that ALS and FTD share a dopaminergic disturbance in serum, while these changes are also partly present in CSF. This could possibly be caused by dopaminergic terminal dysfunction in (cortical) projection areas of the SN and VTA. The compounds of the KP did not appear to be altered in FTD or ALS.

## Electronic supplementary material

Below is the link to the electronic supplementary material.

Supplementary file1 (DOCX 15 kb)

Supplementary file2 (DOCX 18 kb)
