# Supplementary material for: Monoaminergic and Kynurenergic Characterization of Frontotemporal Dementia and Amyotrophic Lateral Sclerosis in Cerebrospinal Fluid and Serum
Source: Neurochem Res. 2020 Mar 4;45(5):1191–201. doi: 10.1007/s11064-020-03002-5 (PMC7162843; doi:10.1007/s11064-020-03002-5)
Supplement: Supplementary file 1 — Supplementary file1 (DOCX 15 kb) [file 11064_2020_3002_MOESM1_ESM.docx]

**Monoaminergic and kynurenergic characterization of frontotemporal dementia and amyotrophic lateral sclerosis in cerebrospinal fluid and serum**

**Neurochemical Research**

Jana Janssens^a,b^, Yannick Vermeiren^a,b^, Martijn van Faassen^c^, Claude van der Ley^c^, Ido P. Kema^c^, Peter P. De Deyn^a,b,d,*^.

^a^Department of Biomedical Sciences, Neurochemistry and Behaviour, Institute Born-Bunge (IBB), University of Antwerp, Wilrijk, Belgium.

^b^Department of Neurology, Alzheimer Center Groningen, University Medical Center Groningen (UMCG) and University of Groningen, Groningen, The Netherlands.

^c^Department of Laboratory Medicine, University Medical Center Groningen (UMCG) and University of Groningen, Groningen, The Netherlands.

^d^Faculty of Medicine and Health Sciences, University of Antwerp, Wilrijk, Belgium.

^*^Corresponding author: Prof. Dr. Peter P. De Deyn, Department of Neurology and Alzheimer Center, University Medical Center Groningen (UMCG), Hanzeplein 1, 9713 GZ Groningen, The Netherlands, e-mail: [p.p.de.deyn@umcg.nl](javascript:redir('umcg.nl'%20,%20'p.p.de.deyn');); telephone number: +3150 361 2401+3150 361 2401

**Online Resource 1. Types of medication across diagnostic categories.**

| **Parameter** | **Compounds of interest** | **Sample type** | **CONTR** | **FTD** | **FTD-ALS** | **ALS** | **Test statistic** |
| --- | --- | --- | --- | --- | --- | --- | --- |
| Taking/not taking psychotropic medication | Monoamines | CSF | 9/7 | 25/9 | 0/1 | 4/13 | Fisher’s Exact=12.6;  *P*<0.05 |
|  |  | Serum | 10/6 | 25/9 | 0/1 | 4/12 | Fisher’s Exact=11.8;  *P*<0.05 |
|  | Kynurenines | CSF | 9/7 | 25/9 | 0/1 | 4/13 | Fisher’s Exact=12.6;  *P*<0.05 |
|  |  | Serum | 9/7 | 25/9 | 0/1 | 4/12 | Fisher’s Exact=11.6;  *P*<0.05 |
| Taking/not taking riluzole | Monoamines | CSF | 0/16 | 0/34 | 0/1 | 7/10 | Fisher’s Exact=18.4;  *P*<0.05 |
|  |  | Serum | 0/16 | 0/34 | 0/1 | 7/9 | Fisher’s Exact=19.2;  *P*<0.05 |
|  | Kynurenines | CSF | 0/16 | 0/34 | 0/1 | 7/10 | Fisher’s Exact=18.4;  *P*<0.05 |
|  |  | Serum | 0/16 | 0/34 | 0/1 | 7/9 | Fisher’s Exact=19.2;  *P*<0.05 |
| Taking/not taking VitB | Monoamines | CSF | 0/16 | 7/27 | 0/1 | 5/12 | Fisher’s Exact=6.1;  *P*>0.05 |
|  |  | Serum | 0/16 | 7/27 | 0/1 | 5/11 | Fisher’s Exact=6.5;  *P*>0.05 |
|  | Kynurenines | CSF | 0/16 | 7/27 | 0/1 | 5/12 | Fisher’s Exact=6.1;  *P*>0.05 |
|  |  | Serum | 0/16 | 7/27 | 0/1 | 5/11 | Fisher’s Exact=6.5;  *P*>0.05 |

Abbreviations: ALS: amyotrophic lateral sclerosis; CONTR: control; CSF: cerebrospinal fluid; FTD: frontotemporal dementia; FTD-ALS: frontotemporal dementia – amyotrophic lateral sclerosis; VitB: vitamin B.
