# Supplementary material for: Monoaminergic and Kynurenergic Characterization of Frontotemporal Dementia and Amyotrophic Lateral Sclerosis in Cerebrospinal Fluid and Serum
Source: Neurochem Res. 2020 Mar 4;45(5):1191–201. doi: 10.1007/s11064-020-03002-5 (PMC7162843; doi:10.1007/s11064-020-03002-5)
Supplement: Supplementary file 2 — Supplementary file2 (DOCX 18 kb) [file 11064_2020_3002_MOESM2_ESM.docx]

**Monoaminergic and kynurenergic characterization of frontotemporal dementia and amyotrophic lateral sclerosis in cerebrospinal fluid and serum**

**Neurochemical Research**

Jana Janssens^a,b^, Yannick Vermeiren^a,b^, Martijn van Faassen^c^, Claude van der Ley^c^, Ido P. Kema^c^, Peter P. De Deyn^a,b,d,*^.

^a^Department of Biomedical Sciences, Neurochemistry and Behaviour, Institute Born-Bunge (IBB), University of Antwerp, Wilrijk, Belgium.

^b^Department of Neurology, Alzheimer Center Groningen, University Medical Center Groningen (UMCG) and University of Groningen, Groningen, The Netherlands.

^c^Department of Laboratory Medicine, University Medical Center Groningen (UMCG) and University of Groningen, Groningen, The Netherlands.

^d^Faculty of Medicine and Health Sciences, University of Antwerp, Wilrijk, Belgium.

^*^Corresponding author: Prof. Dr. Peter P. De Deyn, Department of Neurology and Alzheimer Center, University Medical Center Groningen (UMCG), Hanzeplein 1, 9713 GZ Groningen, The Netherlands, e-mail: [p.p.de.deyn@umcg.nl](javascript:redir('umcg.nl'%20,%20'p.p.de.deyn');); telephone number: +3150 361 2401+3150 361 2401

**Online Resource 2. Concentrations of statistically significant monoamines/ kynurenines, metabolites and ratios.**

| **Parameter** | **CONTR** | **FTD** | **FTD-ALS** | **ALS** | **Test statistic** |
| --- | --- | --- | --- | --- | --- |
| **CSF MHPG (ng/mL)** | 14.2 (7.7)^a,b^  n=26 | 21.0 (13.2)^a^  n=39 | 25.3 (26.5)  n=4 | 19.5 (7.8)^b^  n=23 | ^a^U=298.0  *P*<0.05  ^b^U=190.0  *P*<0.05 |
| **CSF DOPAC (ng/mL)** | 1.9 (1.6)^a,b^  n=26 | 1.4 (0.6)^a^  n=39 | 1.0 (8.8)  n=4 | 1.2 (0.4)^b^  n=23 | ^a^U=307.5  *P*<0.05  ^b^U=173.0  *P*<0.05 |
| **CSF DA (ng/mL)** | 0.4 (0.1)^a^  n=21 | 0.5 (0.1)^a,c^  n=35 | 0.5  n=2 | 0.4 (0.2)^c^  n=17 | ^a^U = 136.0  *P* < 0.0001  ^c^U = 161.5  *P* < 0.05 |
| **CSF DOPAC/DA** | 4.2 (5.4)^a^  n=21 | 2.8 (1.6)^a^  n=35 | 1.7  n=2 | 3.2 (3.7)  n=17 | ^a^U = 167.0  *P* ≤ 0.001 |
| **CSF HVA/DA** | 101.2 (75.6)^a^  n=21 | 72.1 (93.3)^a^  n=35 | 44.4  n=2 | 106.8 (59.5)  n=17 | ^a^U=221.0  *P*<0.05 |
| **Serum DOPAC (ng/mL)** | 6.9 (4.2)^a,b^  n=26 | 3.8 (1.5)^a^  n=39 | 3.4  n=3 | 4.0 (1.3)^b^  n=20 | ^a^U=265.0  *P*<0.05  ^b^U=140.0  *P*≤0.001 |
| **Serum DA**  **(ng/mL)** | 0.9 (0.3)^a,b^  n=26 | 1.1 (0.3)^a^  n=35 | 1.0  n=3 | 1.1 (0.2)^a,b^  n=20 | ^a^U=200.5  *P*<0.001  ^b^U=115.5  *P*≤0.001 |
| **Serum DOPAC/DA** | 6.9 (7.1)^a,b^  n=26 | 3.6 (1.8)^a^  n=35 | 3.2  n=3 | 3.8 (1.0)^b^  n=20 | ^a^U=181.0  *P*<0.001  ^b^U=100.0  *P*<0.001 |
| **Serum HK/XA** | 3.7 (4.1)  n=24 | 4.4 (4.8)^c^  n=38 | 4.3  n=3 | 2.7 (1.7)^c^  n=18 | ^c^U=173.0  *P*<0.05 |

Data are represented as median with interquartile range between brackets. Differences in monoamines, metabolites or ratios between disease groups remaining statistically significant after Benjamini-Hochberg correction, are depicted by superscript letters a, b and c for CONTR versus FTD, CONTR versus ALS and FTD versus ALS, respectively. Abbreviations: ALS: amyotrophic lateral sclerosis; CONTR: control; CSF: cerebrospinal fluid; DA: dopamine; DOPAC: 3,4-dihydroxyphenylacetic acid; FTD: frontotemporal dementia; FTD-ALS: frontotemporal dementia – amyotrophic lateral sclerosis; HK: 3-hydroxykynurenine; HVA: homovanillic acid; XA: xanthurenic acid.
